# Vision for Perception and Vision for Action in Space Travelers

**DOI:** 10.3389/fphys.2022.806578

**Published:** 2022-03-11

**Authors:** Valeriia Yu. Karpinskaia, Ekaterina V. Pechenkova, Inna S. Zelenskaya, Vsevolod A. Lyakhovetskii

**Affiliations:** ^1^Laboratory of Neurovisualization, N.P. Bechtereva Institute of the Human Brain (Russian Academy of Sciences), St. Petersburg, Russia; ^2^Laboratory for Cognitive Research, HSE University, Moscow, Russia; ^3^Laboratory of Gravitational Physiology of the Sensorimotor System, Institute of Biomedical Problems, Russian Academy of Sciences, Moscow, Russia; ^4^Laboratory of Movement Physiology, Pavlov Institute of Physiology, Russian Academy of Sciences, St. Petersburg, Russia

**Keywords:** microgravity, dorsal pathway, ventral pathway, perception, action

## Introduction

Microgravity challenges the human body and brain in many different ways. One of the most evident challenges is the altered functioning of sensory systems, including absent support afference, degraded proprioceptive feedback and unreliable vestibular input. The result is a conflict between the input from different channels, which is resolved through sensory realignment and reweighting (Horak et al., 1990; Block and Bastian, 2012). Vision remains informative and gains priority in the planning and feedback for locomotion and manual actions during spaceflight (Berger et al., 1997). The greater role of visual input in sensorimotor coordination is usually analyzed in the context of compensatory strategies or sensory reweighting and in comparison with other sensory modalities (Kornilova and Kozlovskaya, 2003; Clément, 2007). Alterations and adaptations within the visual system per se, including the transformed interplay of its subsystems, has not been the focus of previous analyses. As we will demonstrate below, experimental studies of the changes in sensorimotor coordination during spaceflight or in ground-based microgravity models usually address performance in specific visually guided motor tasks rather than more basic neurocognitive mechanisms of the adaptive processes.

We suggest that the theory of two visual systems (Goodale and Milner, 1992; Rizzolatti and Matelli, 2003; Kravitz et al., 2011) is a promising framework for facilitating the understanding of sensorimotor coordination in space. Two streams, or pathways, in the neural processing of visual information, both originate in the primary visual cortex (Mishkin and Ungerleider, 1982). The ventral stream (the “what” pathway) ascends to the anterior part of the temporal lobe and provides information for visual awareness, or conscious perception (the “vision for perception” system). Meanwhile, the dorsal stream (the “where” or “how” pathway) ascends to the parietal lobe and propagates to the premotor cortex; it is considered to be the “vision for action” system since it is involved in the processing of spatial information critical for visually guided actions such as object and tool manipulation, and it is believed to be immune to visual illusions and to function independently of subjective states of consciousness (Milner and Goodale, 1995; Giese and Rizzolatti, 2015).

In this opinion paper, we will briefly review the facts that are potentially relevant for predicting alterations in the ventral and dorsal pathways during the actual spaceflight and in ground-based microgravity analogs. We will also discuss the idea that the dorsal and ventral streams may be affected differently during a microgravity-stimulated recalibration of sensorimotor coordination.

## Ventral Pathway in Microgravity

The ventral visual subsystem's primary function is the recognition and identification of visual objects and events; it is believed to incorporate visual memory representations of object categories, individual objects, and features such as color and shape, and to utilize an allocentric frame of reference (Norman, 2002; Jang and Jang, 2018). The ventral stream is recruited in tasks that require verbal judgments on visual stimuli (Milner, 2017). Findings relevant to these aspects of visual perception may be used as markers of the functioning of the ventral stream.

Scarce evidence suggests that microgravity may induce subtle changes in color perception, mainly in perceived brightness and saturation (Schlacht et al., 2009 for review). The recognition of complex objects such as faces remains generally preserved. The absence of a gravitational frame of reference and the “upright” direction in the spaceflight environment has implications for object recognition since the crew is frequently exposed to unusual points of view of objects, including upside-down perspectives. One possible adaptation may be the development of viewpoint-invariant recognition abilities. This could lead to a disappearance of the inversion effect in face recognition (poor recognition of upside-down faces, popularized as the Thatcher illusion). However, the inversion effect is preserved, as has been revealed on the Mir space station. Furthermore, the memory for novel faces suffers during the flight compared to pre- and post-mission measurements, which indicates difficulties in the ventral stream rather than its hyperfunctioning. So, if they exist at all, viewpoint-invariant representations developed during a spaceflight are limited to familiar objects seen from different viewpoints on a daily basis (for a review, see Leone, 1998).

## Dorsal Pathway in Microgravity

The primary function of the dorsal stream is the visual guidance of behavior (Norman, 2002). Frequently studied special cases include manual actions performed with stationary visual targets (pointing, aiming, reaching, and grasping) or with moving objects (tracking and catching). The dorsal subsystem incorporates spatial representations such as the 3D structure of objects and spatial relations, and it enables mental spatial manipulations such as rotation (Freud et al., 2016); it processes dynamic features such as motion direction or speed and is able to utilize an egocentric frame of reference (Norman, 2002). The dorsal pathway is recruited for visuomotor control (Milner, 2017). Evidence on these aspects of perception and action may be used as markers of the functioning of the dorsal visual stream.

The characteristics of visually guided movements in microgravity are task-dependent (Bock et al., 2003). During space missions, pointing (with variations such as aiming and step-tracking) has been found to slow down without any loss of accuracy (Berger et al., 1997; Sangals et al., 1999; Mechtcheriakov et al., 2002; Bock et al., 2003; Casellato et al., 2016); the same is true for grasping (Bock et al., 2003). In some Mir station crew members (but not in others), the duration of movements recovered in a couple of months after the launch. However, the presence or absence of visual feedback affects pointing accuracy but not the velocity profile of the movement. Microgravity, in contrast, did not affect pointing accuracy, but it decreased peak velocity and acceleration (Berger et al., 1997; Mechtcheriakov et al., 2002). Converging evidence from Neurolab experiments was reported by Bock et al. (2001, 2003). Therefore, the transformation of pointing actions cannot be attributed to specific adaptations of the dorsal visual pathway. Data from parabolic flights are contradictory [aiming slowed down (Crevecoeur et al., 2010, 2014), while reaching was unaffected (Gaveau et al., 2016; Macaluso et al., 2017)], which indicates that the adaptation of the velocity profile needs time to unfold.

The hand movements involved in catching a ball are initiated earlier during a space mission than in terrestrial conditions. This suggests that, even in weightlessness, humans use an internal model of the Earth's gravity rather than relying on a Gibsonian direct perception when anticipating object motion (McIntyre et al., 2001). A dedicated fMRI study by Indovina et al. (2005) showed that using such internal models in normogravity may be implemented as an interplay between dorsal pathway areas (such as MT) and the vestibular neural network. The internal gravity model may be adjusted by training using mental imagery of an object's motion in space station vs. terrestrial environments (Gravano et al., 2021).

For manual tracking, the data are inconsistent; some studies find it slowing down during a space mission (Manzey et al., 2000) and others report that it is unaffected (Bock et al., 2003). Decrements in manual tracking performance may be driven by microgravity effects per se, most prominently during the early adaptation phase (accuracy may recover in about 3 weeks), and by attentional impairment due to stress and fatigue later in the mission (Manzey et al., 2000). Consistent with this, Kornilova et al. (2016) showed that optokinetic stimulation improves both visual and manual tracking in a dry immersion microgravity model, supposedly by recruiting more attentional resources (which may also engage the dorsal stream as a neural substrate of attention; Corbetta and Shulman, 2002).

Adaptation to the space station environment with its absence of the gravitational vertical and the presence of a variety of viewpoints for all surroundings may involve a boost in the traveler's mental rotation ability (Leone, 1998), which again implies the activation of the dorsal pathway. The available evidence suggests that this ability remains unaltered (Leone et al., 1995) or may indeed be slightly improved (Matsakis et al., 1993). Transient microgravity in parabolic flights does not change mental rotation abilities in an object-based reference frame (Grabherr et al., 2007; Dalecki et al., 2012), but inconclusive evidence exists for the egocentric reference frame (impaired: Grabherr et al., 2007; unchanged: Dalecki et al., 2012; improved: Meirhaeghe et al., 2020). Such discrepancies may reflect dynamic adaptation in the dorsal stream upon entering the microgravity environment.

Data on the egocentric reference frame also suggest the active adaptation of the dorsal stream to microgravity. When asked to reproduce a reference line from memory by adjusting a test line's tilt, participants on Earth estimated the vertical and horizontal lines with greater accuracy than other orientations, but only for the upright (vs. tilted) body position. In space, the horizontal or vertical line superiority persisted, but within the egocentric rather than the allocentric reference frame (Lipshits and McIntyre, 1999; McIntyre and Lipshits, 2008). At the same time, Watt (1997) showed that maintaining an accurate egocentric spatial map may be challenging in microgravity. Cheron et al. (2014) found electrophysiological evidence for the transformation of top-down signals within the visual system during a navigational task in virtual reality aboard the ISS, suggesting a reorganization of the dorsal pathway. Although they are each subtle, dorsal stream modifications during the flight may become crucial cumulatively, since astronauts demonstrate a drastic decline in the ability to drive a vehicle soon after re-entry to Earth (Moore et al., 2019).

## Discussion

The available evidence from numerous studies on visuomotor coordination in space hardly leads to a systematic inference about the functioning of the ventral and dorsal streams during spaceflight. The active use of the egocentric frame of reference in microgravity suggests an increased role of the dorsal visual stream. This hypothesis is in general agreement with other suggestions, but to test it directly, access to dissociable measures of the two streams' functioning is needed. The most straightforward approach requires neurophysiological equipment. For instance, EEG-based ERPs may be used to describe the complex dynamics of parietal cortex adaptation to ground-based microgravity models (Wang et al., 2017). Robust behavioral measures have been developed within the perception-action framework. Dissociation may be observed through a comparison of the drawing of an actual object (model) vs. copying the object from another drawing or reproducing it from memory (Milner and Goodale, 1995). Another technique involves a comparison of the ventral-originated representation (accessed through verbal report) and the dorsal-originated representation (accessed through motor output) of geometric properties such as size, distance and tilt. Dissociation between the two types of estimations may be reliably found for geometrical-optical illusions. The between-finger distance in a hand about to grasp an object may accurately reflect its size (dorsal-stream estimation), while verbal judgments of the same object's size (ventral-stream estimation) may be prone to illusions (Aglioti et al., 1995). The same logic may be used for pointing and tracking (Bruno et al., 2008; Stöttinger et al., 2012) to estimate the activation of the two visual subsystems and their balance in microgravity.

Drawings and geometrical illusions have already been evaluated in microgravity conditions, but only as representational drawings (i.e., with eyes closed) for the former and only with verbal estimations for the latter. Villard et al. (2005) showed that the strength of illusions that involve a misinterpretation of depth (the inverted-T; horizontal Muller-Lyer; Ponzo; and Hering illusions) significantly decreased during a parabolic flight. Only the inverted-T illusion, but not the Ponzo or vertical Muller-Lyer illusion, decreased in strength during a long-term space flight aboard the ISS (Clément et al., 2012). In line with these findings, the ambiguity of perceptions of depth-reversible drawings, such as the Necker cube, gradually increased in a long-term space flight, while in terrestrial conditions the same observers tended to have a preferred interpretation that they saw more often than the other interpretation (Clement et al., 2015). However, these results provide no information on the interplay of the two visual subsystems since the dissociation requirement was not met and only the ventral stream output was tested.

A dissociation between the verbal and motor estimations of the Ponzo and Muller-Lyer illusions was tested in dry immersion conditions, and both were found to be sensitive to the absence of support afference (Sosnina et al., 2019; Sosnina I. et al., 2021; Sosnina I. S. et al., 2021). In the proposed paradigm, participants are presented with the illusions or a neutral pair of lines of the same length on a touch screen; they are asked either to give a verbal estimation of which line section is longer and by what percentage, or to provide a motor estimation by sliding their finger over each line (Karpinskaia et al., 2016; Lyakhovetskii and Karpinskaia, 2017). We believe that the further development of this line of research, beginning with a careful analysis of the illusions and the different estimation tasks in ground-based models and further bringing the ventral vs. dorsal visual stream dissociation experiments to space, will significantly contribute to our knowledge on perception and action in space travelers.

## Author Contributions

VK and VL contributed to the conception of the paper. VK and EP performed the literature review and wrote the manuscript. VL and IZ contributed to the discussion. All authors contributed to the manuscript's revision and read and approved the submitted version.

## Funding

This research was supported by the Russian Science Foundation Grant # 19-15-00435.

## Conflict of Interest

The authors declare that the research was conducted in the absence of any commercial or financial relationships that could be construed as a potential conflict of interest.

## Publisher's Note

All claims expressed in this article are solely those of the authors and do not necessarily represent those of their affiliated organizations, or those of the publisher, the editors and the reviewers. Any product that may be evaluated in this article, or claim that may be made by its manufacturer, is not guaranteed or endorsed by the publisher.
